# Prevalence and correlation with sex, age, and dental status of bone apposition at the mandibular angle and radiographic alterations of the temporomandibular joints: a retrospective observational study in an adult Swiss population

**DOI:** 10.1186/s12903-024-03855-0

**Published:** 2024-02-06

**Authors:** Michelle Simonek, Jens Christoph Türp, Michael M. Bornstein, Dorothea Dagassan-Berndt

**Affiliations:** 1 Department of Oral Surgery, University Center for Dental Medicine Basel (UZB), University of Basel, Basel, Switzerland; 2 Division of Temporomandibular Disorders and Orofacial Pain, Department of Oral Health & Medicine, University Center for Dental Medicine Basel (UZB), University of Basel, Basel, Switzerland; 3 Department of Oral Health & Medicine, University Center for Dental Medicine Basel (UZB), University of Basel, Basel, Switzerland; 4 Center for Dental Imaging, University Center for Dental Medicine Basel (UZB), University of Basel, Basel, Switzerland

**Keywords:** Mandible, Temporomandibular joint, Bone remodeling, Bone apposition, Panoramic radiography

## Abstract

**Background:**

The purpose of this study was to determine the prevalence of radiographic changes in the mandibular angle (bone apposition) and osseous alterations in the temporomandibular joints (TMJs) in the adult population of Switzerland. In addition, the study intended to investigate possible correlations between the two sites of contour bone changes (mandibular angle and TMJ) and to analyze various patient-related factors, including sex, age, dental status, and medical history.

**Methods:**

Panoramic radiographs of 600 patients distributed into six age groups (283 females, 317 males, aged 20 to 79 years) were included to evaluate radiographic changes. The bone in the mandibular angle region and the shape of the condylar heads were examined for contour changes (bone apposition at the jaw angles and osseous changes of the TMJs). General estimating equations, binormal tests, and chi-squared tests were used for statistical analysis.

**Results:**

Approximately half of the mandibular angles (47.8%) showed bone apposition, mostly bilateral. TMJ alterations were less common (27%), often unilateral, with flattening being the most frequent finding. No significant correlation was found between the two sites. Bone apposition at the mandibular angle showed a significant male predominance, whereas TMJ changes did not differ by sex. Alterations in both sites increased with age, and were not related to dental status or analgesic use.

**Conclusions:**

Bone apposition at the mandibular angle should be interpreted as part of the natural functional adaptation of the bone associated with aging. Assuming that parafunctional habits may influence the development and progression of alterations in the mandibular angle or TMJs, the presence of radiographic changes in these areas should prompt dental clinicians to investigate further in this direction.

**Trial registration:**

The study was approved by the Swiss Association of Research Ethics Committees (swissethics), BASEC reference number: 2020–00963 (25.05.2020).

## Background

In daily clinical practice, osseous changes of the mandibular angle and the temporomandibular joint (TMJ) can be detected on panoramic radiographs. Since panoramic radiography provides valuable, relatively low-dose [1, 2] visualization of the bony and dental structures of the jaw, it is often well suited for initial radiographic imaging in various clinical scenarios.

Repetitive tensile and compressive loads resulting from muscle contraction have long been recognized as factors that induce physiological bone adaptations. These adaptations typically involve bone resorption by osteoclasts in response to compressive loading and bone formation by osteoblasts in response to tensile loading [3–5]. In dental medicine, the concept of tissue remodelling is well known from the biological mechanisms of orthodontic tooth movement. The compression–tension theory is widely aknowledged and proposes that cellular responses are modulated in response to mechanical stress (orthodontic force) applied to the periodontal ligament (PDL) and alveolar bone. This cellular strain, involving stretching or compression of PDL-cells and osteocytes, directly triggers the activation of osteoclasts and osteoblasts. Consequently, this leads to bone resorption in the compression side and bone deposition at the tension side of the moved tooth [5, 6]. Furthermore, osseous changes at the mandibular angle region and in the TMJs have been reported in the literature, particularly among patients with dental parafunctions. Studies have documented changes of the mandibular bone in the jaw angle region in patients with bruxims [7–12]. Similarly, patients with temporomandibular joint osteoarthritis present progressive cartilage breakdown and degenerative osseous alterations in the TMJs [13–15].

However, osseous changes in both the mandibular angle and the TMJs are often recognized by the clinician as a secondary finding on panoramic radiographs in individuals who do not report symptoms or who have been diagnosed with bruxism. Therefore, the present investigation was designed to focus on the general population to assess osseous changes, without specifically selecting patients with diagnosed dental parafunctions. The primary objective of this study was to determine the prevalence of mandibular angle bone apposition and TMJ alterations. Secondary objectives were to evaluate potential correlations between alterations at the two sites, as no such data are available in the literature. Additionally, patient-related factors such as sex, age, dental status and medical history were analyzed.

## Methods

### Study population

Six hundred panoramic radiographs of adult patients (over 20 years of age) were randomly selected from the dental imaging archive of the University Center for Dental Medicine Basel (UZB). They represented a cross-section of the general adult population in Switzerland. The radiographs were taken between September 2018 and March 2020 from patients scheduled for regular dental diagnosis and treatment planning purposes. Cranex™ D (Soredex, Tuusala, Finland) or PaX-i Orangedental (Vatech, South Korea) were used for imaging, with an indicated magnification factor of 1:1.25 (Cranex™ D) and 1:1 (PaX-i). Digora version 2.9 software (Soredex by Kavo Kerr Group) was used to visualize the images without applying any additional filtering.

The study population was divided into six age groups (20–29, 30–39, 40–49, 50–59, 60–69, and 70–79 years). With 100 subjects in each group, the study population consisted of 283 women and 317 men. The gender distribution was nearly equal across the age groups. To be eligible for the study, the TMJs and the mandibular angles had to be completely depicted on the radiographs.

### Exclusion criteria

Exclusion criteria were as follows:

- subjects with known bone metabolic disorders (e.g., osteoporosis) and/or taking corresponding medications (e.g. bisphosphonates, denosumab);
- subjects with incomplete medical history or lack of informed consent;
- individuals with ongoing orthodontic therapy;
- radiographs with incomplete visualization of the mandibular angles or TMJs;
- non-orthogonal radiographs (including severe retroflexion, severe anteflexion, and incorrect sagittal alignment).

A total of 206 subjects were excluded during the selection process based on the above criteria.

### Evaluation of radiographs

**Fig. 1:**
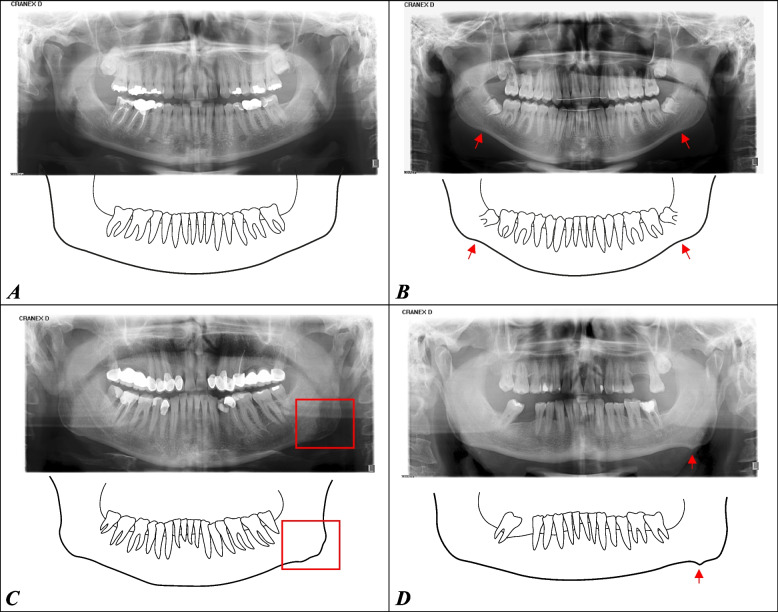
Radiological evaluation of bone apposition at the mandibular angle and grade classification: A and B are categorized as “no alteration”. **A** shows the natural convexity of the mandibular contour without directional deviation, **B** describes a standard variant of the mandibular angle with directional deviation but without bone apposition. **C** and **D** show examples of bone apposition in combination with directional deviation (here: generalized apposition with inhomogeneous surface (C) and localized spike-shaped apposition at one or more sites (D))

All radiographs were reviewed by a single principal investigator (MS). Each mandibular angle of the 600 patients was analyzed separately. Prior to analysis, the principal investigator underwent a three-step calibration process to establish a classification system for the radiographic findings (during this process, two other reviewers [DD, JCT] examined 10% of the radiographs on two separate occasions, and the findings were reconciled). Previously published research on bone apposition at the mandibular angles of bruxism patients [7] was used as a reference to determine the presence or absence of bone apposition for each mandibular angle. Subsequently, each of the 1200 mandibular angles was individually assigned to one of four grades (Fig. 1 A-D) according to the shape of their basal cortical bone. Macroscopically visible bone formations were categorized as either unilateral or bilateral. Indeterminate bone apposition, single direction deviation, or very mild manifestations on the mandibular angles were classified as “no alteration”.

**Fig. 2:**
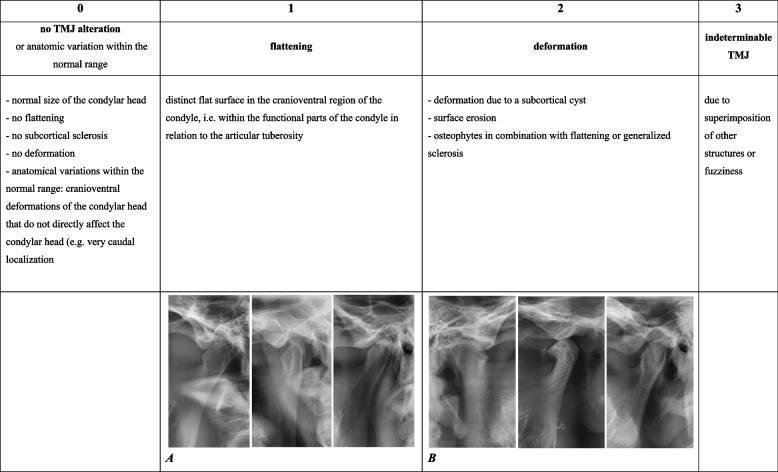
Stages 0 to 3 of TMJ alterations and some corresponding radiographic examples (*A* and *B*)

TMJ grading was based on the image analysis criteria of the Research Diagnostic Criteria for Temporomandibular Disorders (RDC/TMD) [16] to classify each TMJ for the presence or absence of condylar changes ranging from no change to functional deformity (stages 0 to 2), as shown in Fig. 2. Some TMJs were “unrateable” due to superimposition of other structures or fuzziness on either the left or right side. After excluding them (*n* = 56 TMJs from 46 subjects) from further statistical analysis, this resulted in a total of 1108 TMJs that could be used for further evaluation (554 subjects) and a total of 1200 mandibular angles (600 subjects).

Dental status was assessed using the Eichner index [17] to classify the dental situation of the study participants into three classes:

1) fully dentate jaw (excluding third molars);

2) partially dentate jaw (one or more teeth missing);

3) edentulous jaw.

The use of analgesics was recorded on the basis of self-reported data from the patients’ medical history forms. Bruxism or other parafunctions were not systematically assessed; however, if bruxism was present and diagnosed, it was noted and considered in the subsequent evaluation.

### Statistical analysis

First, all data were analyzed descriptively. TMJ alterations that were grouped as indeterminable on either the left or right side were excluded from further analysis. Also, due to the small number of events detected for TMJ deformities, TMJ findings were regrouped into binary variables (healthy vs pathological [flattening/ deformation]) for the statistical analyses, unless otherwise noted. Because of the high intraclass correlation of the binary outcomes (TMJ or mandibular changes) within the same individual, a generalized estimating equation model with logit link function and unstructured correlation assumption for the repeated measures (on the left and right side in the same individual) was used to explore the association between the outcome and other factors individually. The binormal test was performed to investigate whether there was a statistically significant difference in the distribution of unilateral and bilateral pathological changes. Chi-squared tests were used to explore associations between the outcome and other categorical variables for each side individually. For the present statistical analysis, *p*-values less than or equal to 0.05 were considered as statistically significant. All results were calculated with the statistical software package SPSS (version 28, IBM Corp., Armonk, NY).

## Results

Approximately half of the mandibular angles showed bone apposition (574/1200, 47.8%), while TMJ pathology was less common (flattening in 288 cases [24%], severe deformity in 33 [2.7%]) (Table 1). This study examined whether these changes were unilateral or bilateral. The results showed that bone apposition at the mandibular angle had a significantly higher proportion of bilateral occurrence (71%) (*p* < 0.001). Conversely, TMJs with ‘flattening’ (61.6%) (*p* = 0.001) or ‘deformity’ (76%) (*p* = 0.015) had a higher proportion of unilateral occurrences. Regarding a possible association between mandibular angles (no vs. apposition) and TMJs (healthy vs. pathology), no significant correlation was generally observed (*p* = 0.724). Separate analysis of the right and left sides also showed no significant association (chi-square test: left: *p* = 0.472; right: *p* = 0.975). Even in the absence of any TMJ alteration, bone apposition at the mandibular angle occasionally occurred (Table 2).

**Table 1:**
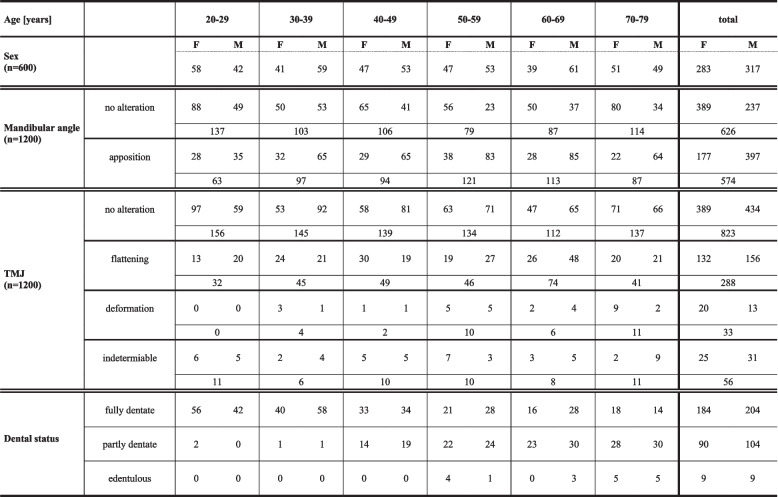
Prevalence of mandibular angle bone apposition and TMJ alterations according to sex, age, and dental status

**Table 2:** Relationship between mandibular angle changes and the ipsilateral TMJ manifesting radiologic changes in each side of the 554 patients

			Mandibular angle (n=1108^a^)				*p*-value
			left (*n*=554)		right (*n*=554)		
			no alteration	apposition	no alteration	apposition	
**TMJ (n=1108**^a^**)**	**left (n=554)**	no alteration	205 (72.4%)	194 (71.6%)			left:0.823 right:0.232
		pathology (flattening/deformation)	78 (27.6%)	77 (28.4%)			
	**right (n=554)**	no alteration			225 (75.0%)	179 (70.5%)	
		pathology (flattening/deformation)			75 (25.0%)	75 (29.5%)	

^a^after excluding subjects with indeterminable TMJs (*n*=46) for correct comparison

In the statistical analysis, sex was a significant factor for changes of the mandibular angle (no vs. apposition) (*p* < 0.001) with males being more likely to exhibit bone apposition (OR = 3.72, 95% CI: 2.71–5.13), but was a non-significant factor for TMJ alterations (healthy vs. pathologic) (*p* = 0.893).

Age showed significance for both bone apposition (*p* = 0.001) (chi-square test: left: *p* < 0.001; right: *p* = 0.010) and TMJ pathology (*p* = 0.001) (chi-square test: left: *p* = 0.027; right: *p* < 0.001). The younger age group (20–29 years) was less likely to have bone apposition than the middle age group (50–69 years), and also less likely to have TMJ pathology than the 60–69 years age group (*p* < 0.001).

When assessing other patient-related factors, such as dental status, the majority of individuals were fully dentate (388; 64.7%), approximately one third were partially dentate (194/600), and a small percentage (18; 3%) were edentulous. In general, tooth loss became more prevalent with increasing age (Table 2). No significant differences in dental status were observed based on sex. There was also no significant association between dental status and bone apposition (*p* = 0.592) or TMJ alteration (*p* = 0.217).

Bruxism was confirmed in only 22 subjects, of whom 16 had bone apposition in at least one mandibular angle and 10 had condylar changes (flattening or deformation).

Regarding analgesic use, only 10% of the patients reported regular intake. Of these, only few (10%) had bony changes in the TMJs or mandibular angles (Table 3).

**Table 3:** Use of analgesics and contaminating changes in the TMJs or mandibular angle by age and sex

Group		Intake of analgesics	Contaminating changes of the TMJ or mandibular angle
**Age [years]**	20-29	5	2
	30-39	5	4
	40-49	16	15
	50-59	12	8
	60-69	13	10
	70-79	11	10
**Sex**			
** F**	*n*=283	27	18
		(9.5%)	(6.4%)
** M**	*n*=317	35	31
		(11%)	(9.8%)

## Discussion

In the present study, a total of 600 panoramic radiographs were analyzed from patients in six different age groups, ranging from 20 to 79 years. Both, the mandibular angles and the TMJs were examined individually. The study focused exclusively on adult participants, because a previous investigation reported no bone apposition at the mandibular angle in adolescents [7]. Furthermore, TMJ pathology is generally unlikely to be observed in adolescents due to the relatively short duration of loading and the fact that osteoarthritis in other joints typically develops at a later age (27 years for the knee and 59 years for the foot) [18]. The absence of TMJ deformities observed in our group of young patients, aged 20 to 29 years, is consistent with these previous findings.

The current investigation reports a prevalence of 47.8% for mandibular bone apposition in the randomly selected population. Other studies describe mandibular bone apposition in patients diagnosed with bruxism: Türp et al. (2021) reported the same percentage of 47.5% based on a sample of 100 panoramic radiographs [19], while Hayek et al. (2022) presented a slightly higher prevalence of 52% in a cohort of 150 patients with bruxism [8]. Isman (2021) reported a prevalence of 31.7% for spike-shaped appositions in 60 bruxism patients [9]. Only Casazza et al. (2023) reported higher prevalences of approximately 80% in a small group of bruxism patients [12].

Regarding TMJ alterations, our study found a prevalence of 27%, with flattening being the most common finding (24%). Hiltunen et al. (2002) reported a comparable prevalence of flattening (17%) in a sample of 88 panoramic radiographs of patients without previously diagnosed TMD [20]. In contrast, three-dimensional analysis of cone beam computed tomography (CBCT) scans of patients with degenerative joint disease showed an expectedly higher prevalence of articular flattening (73–77%) [21, 22].

Bone apposition and TMJ alterations appear to develop independently of each other. Bone apposition often occurs without concomitant ipsilateral TMJ changes. Again, no statistically significant correlation was found (*p* = 0.724).

It has been proposed that degenerative TMJ changes result from a gradual accumulation of tissue damage due to a decrease in the cellular adaptive capacity [23]. The international RDC/TMD Consortium Network and the Orofacial Pain Special Interest Group [15] stated that mild TMJ alterations are age-related physiologic processes, but that severe manifestations, such as deformaties, osteophytes, and subchondral cysts, may indicate pathologic conditions. In the present data, changes at both sites showed an increased prevalence with advancing age. Condylar changes and mandibular bone apposition were most prevalent in individuals aged 50 to 69 years. The literature supports the strong correlation between aging and temporomandibular changes [24–27], but age-related findings for mandibular angle bone apposition are lacking. Luder performed autopsies on 25 TMJs and found that degeneration peaked at approximately 55 to 60 years of age, with no significant increase thereafter and a decrease with older age [23]. Ishibashi et al. (1995) also report that the peak of temporomandibular change occurs between the fifties and seventies [28].

There is a consensus that bone formation is associated with increased tensile forces [3–5]. Previous studies have suggested that bone apposition may serve as a radiographic indicator of bruxism, possibly related to the increased loads resulting from excessive masseter and medial pterygoid muscle activity in patients with bruxism [8, 9, 19]. However, the results of this study raise some controversial points regarding this assumption. On the one hand, the fact that three-quarters of the previously diagnosed bruxism patients in this study also had bone apposition in at least one mandibular angle (16 of 22, 73%) supports the hypothesis. On the other hand, the similar, high overall prevalence (47.8%) in the 1200 individuals of the evaluated “general population” compared to the bruxism patient group (47.5%) evaluated by Türp et al. casts doubt on this correlation. Therefore, it is more plausible to consider bone apposition as part of physiological adaptation over time rather than only a pathological condition.

In terms of sex differences, males had a significantly higher prevalence of bone apposition, whereas the proportion of TMJ pathology detected in females and males in this study was equal. The literature on osseous changes (some of which are degenerative) in the TMJs articular surface and sex remains controversial, with findings from clinical investigations [20, 29] as well as some autopsy studies [23, 25, 27, 28, 30–33] reporting either no apparent differences [20, 23, 28, 30, 31] or female [25, 29, 32] or male predominance [27, 33].

Although the present study did not find a significant influence of the dental status on either bone apposition or the development of TMJ pathology, the literature discusses potential influence of bilateral loss of occlusal support on altering condylar morphology and considers it a possible factor in the development of degenerative changes [23, 33, 34]. Other studies have rejected such relationships [27, 35, 36].

### Limitations

Panoramic radiography offers some advantages (low-dose imaging with adequate visibility and cost-effectiveness [1, 2]) over three-dimensional imaging for the evaluation of major osseous TMJ pathology [37–39] and has been recommended as a screening tool for TMJ pathology [16]. However, its usefulness in TMJ assessment is certainly limited and shows low sensitivity in detecting osseous changes compared to the high-resolution multiplanar images of a CBCT scan [39, 40]. In addition, panoramic radiographs can be distorted and superimposed by other structures, such as the zygomatic process [40]. Specifically, this means that the findings from the present study tend to underestimate the prevalence of TMJ alterations.

Bruxism and TMDs are frequently diagnosed conditions (therapy-relevant prevalence of bruxism: approximately 8% [41, 42], of TMDs about 5 to 12% [15]). This leads to another notable limitation of this study: due to the retrospective study design, it is not possible to prove that a person does not have any parafunctional habits without a clinical examination prior to the study. Ultimately, only an examination of each subject in a sleep laboratory could provide reliable evidence of the absence or presence of (often nocturnal and unconscious) parafunctional habits.

## Conclusions

This study emphasizes that sex and age play a significant role in the development of bone apposition at the mandibular angle and that age is a relevant factor for osseous changes in the TMJs. The results of this study do not exclude the possibility of an association between these alterations and bruxism, as parafunctional habits may contribute to the development and progression of osseous changes. However, the authors emphasize that bone apposition at the mandibular angle and TMJ alterations should also be recognized as partial physiologic adaptations of the bone associated with aging, rather than as signs of pathology. As panoramic radiography continues to be used in routine clinical practice, it can serve as a tool to detect major bone changes. The presence of macroscopically visible radiologic changes should prompt the dental clinician to perform a comprehensive examination, especially in cases of clinical suspicion of a dental parafunction.

## Data Availability

The data sets used and/or analyzed during this study are the property of the authors and are available from the corresponding author only upon reasonable request.

## Abbreviations

RDC/TMD: Research Diagnostic Criteria for Temporomandibular Disorders
TMD: Temporomandibular Disorders
TMJ: Temporomandibular joint
CBCT: Cone beam computed tomography
